# Spin-orbit**-**torque-induced magnetic domain wall motion in Ta/CoFe nanowires with sloped perpendicular magnetic anisotropy

**DOI:** 10.1038/s41598-017-02208-y

**Published:** 2017-05-17

**Authors:** Yue Zhang, Shijiang Luo, Xiaofei Yang, Chang Yang

**Affiliations:** 1 School of Optical and Electronic Information, Huazhong University of Science and Technology, Wuhan, 430074 PR China; 2 Department of Physics and Astronomy, Johns Hopkins University, Baltimore, MD 21218 USA; 3 Department of Mathematics, Harbin Institute of Technology, Harbin, 150001 PR China

## Abstract

In materials with the gradient of magnetic anisotropy, spin-orbit-torque-induced magnetization behaviour has attracted attention because of its intriguing scientific principle and potential application. Most of the magnetization behaviours microscopically originate from magnetic domain wall motion, which can be precisely depicted using the standard cooperative coordinate method (CCM). However, the domain wall motion in materials with the gradient of magnetic anisotropy using the CCM remains lack of investigation. In this paper, by adopting CCM, we established a set of equations to quantitatively depict the spin-orbit-torque-induced motion of domain walls in a Ta/CoFe nanotrack with weak Dzyaloshinskii–Moriya interaction and magnetic anisotropy gradient. The equations were solved numerically, and the solutions are similar to those of a micromagnetic simulation. The results indicate that the enhanced anisotropy along the track acts as a barrier to inhibit the motion of the domain wall. In contrast, the domain wall can be pushed to move in a direction with reduced anisotropy, with the velocity being accelerated by more than twice compared with that for the constant anisotropy case. This substantial velocity manipulation by anisotropy engineering is important in designing novel magnetic information devices with high reading speeds.

## Introduction

Since the concept of “race-track storage” was proposed in 2008^1^, current-induced magnetic domain wall motion in nanotracks has attracted wide research interest because of its application potential in next-generation memory-storage devices with advantages such as low dissipation, high reading speed, small size, and large storage density. The basic physical principle underlying such devices is the exchange of angular momentum between the spins of conducting electrons and magnetic moments in the domain wall due to the spin-transfer torque (STT) or spin-orbit torque (SOT). Recently, SOT-driven domain wall motion has attracted wide attention because of its high energy efficiency^2–4^.

Unlike the bulk STT in a single ferromagnetic nanotrack, the SOT dominates in multilayer films, such as heavy metal (HM)/ferromagnet (FM) multilayer^2, 3, 5^ or HM/FM/oxide multilayer with broken inversion symmetry at the interface^4^. The SOT effect in the multilayer system can originate from the spin Hall effect (SHE) of the HM layer. Recently, some intriguing SOT-induced magnetization behaviours have been discovered in HM/FM bi-layers with *sloped* magnetic parameters. For example, Yu *et al*. discovered field-free magnetization switching in a Ta/CoFeB perpendicularly magnetized (PM) film with magnetic anisotropy gradient^6^. They attributed this phenomenon to an effective magnetic field originating from the gradient of the magnetic anisotropy constant.

From a microscopic perspective, the SOT-induced magnetizing process in a HM/FM multilayer is generally related to the motion of the FM domain wall. For example, the domain wall exhibits chirality and an unexpected Néel-typed structure in the HM/FM multilayer with interfacial Dzyaloshinskii–Moriya interaction (DMI)^3, 7^. The direction of the motion of the domain wall is also related to the sign of the DMI constant and to that of the spin Hall angle of the HM^4, 8–10^. In addition, the domain wall also tilts because of strong DMI, which has been experimentally observed^11, 12^ and theoretically proven^9, 13–16^. Additionally, domain wall motion is closely linked to the magnetic anisotropy constant which can be controlled effectively by manipulating the thickness of the film or by being situated in an electric field^17–20^.

Despite recent progress, determining how the domain wall moves in a HM/FM multilayer with the gradient of magnetic anisotropy remains to be elucidated. Yamada *et al*. proposed an effective field induced by the gradient of magnetic anisotropy and believe that this effective field can drive the wall to move^21^. However, the domain-wall motion in a magnetic system with anisotropy gradient has not been investigated using standard cooperative coordinate method (CCM), which is the basic route to quantitatively study the domain wall motion^7, 22^.

In the present work, using CCM, we have deduced the equations to describe the SOT-driven domain wall motion in a HM/FM multilayer with the gradient of magnetic anisotropy and weak DMI. The equations were solved numerically and compared with the results of a micro-magnetic simulation. Based on the derived equations, the effect of magnetic anisotropy gradient on the domain wall motion has been revealed.

## Principle and Methods

### Numerical Calculation based on collective coordinate model

**Figure 1:**
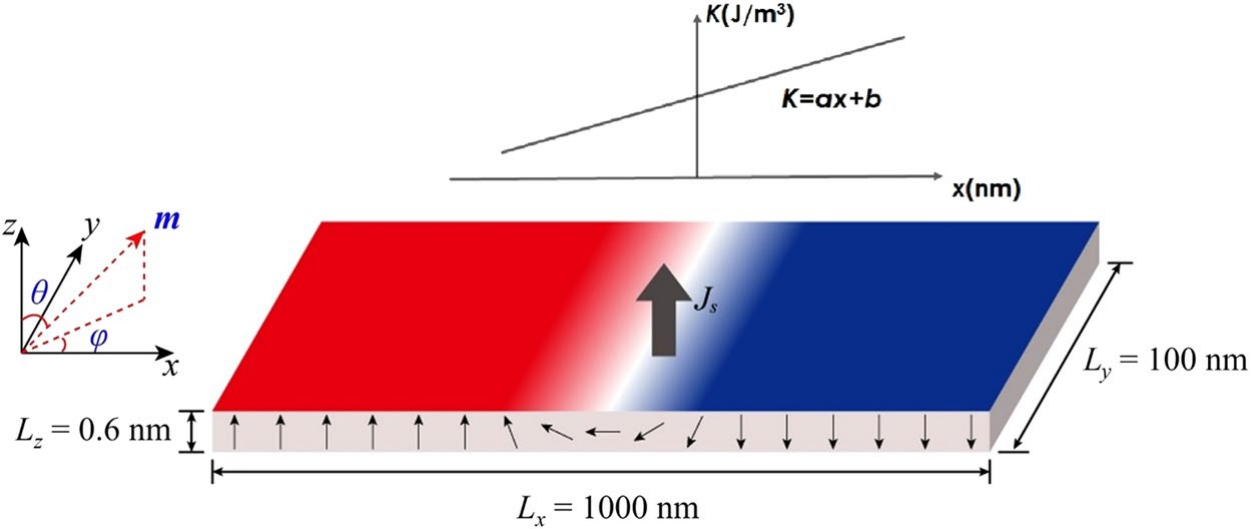
Schematic of SOT-driven domain wall in a nanotrack with magnetic anisotropy gradient. (The theoretic analysis in the paper is based on the up-down domain wall structure in the coordinate system shown in Fig. 1).

To quantitatively describe the dynamics of SOT-driven domain wall motion in a nanotrack with magnetic anisotropy gradient, we develop a one-dimensional (1D) CCM, where the DW is depicted by two collective coordinates: its central position *q* and the azimuthal angle *φ* of the DW magnetization in spherical coordinates, as shown in Fig. 1. Due to the weak DMI, the tilting angle of DW is not taken into consideration here^9^.

The normalized vector for the direction of magnetization is described as $\overrightarrow{m}=(\sin \,\theta \,\cos \,\phi ,\,\sin \,\theta \,\sin \,\phi ,\,\cos \,\theta )$. The polar angle *θ* and the azimuthal angle *φ* marked in Fig. 1 are included in the ansatz for the DW magnetization:

1 $$\theta ={\rm{2arctan}}\{\exp [(x-q)/{\rm{\Delta }}]\},{\rm{and}}\,\phi =\phi (t)$$

where *t* is the time, and

2 $${\rm{\Delta }}=\sqrt{A/(K-{{\rm{\mu }}}_{0}{M}_{S}^{2}/2)},$$

represents the width of the domain wall and *A*, μ_0_, *M*_S_, and *K* are the exchange stiffness constant, vacuum permeability, saturation magnetization, and magnetic anisotropy constant for the PMA film, respectively. In the present work, *K* = *ax* + *b*. This linear function *K*(*x*) is the simplest form for numerical calculation. From the aspect of experiments, to generate PM film with linearly varied *K*, one can fabricate PM film with a gradient of thickness along the track^6^ or by poling piezoelectric substrate in a wedge shape^21^. To determine the parameters *a* and *b* in the function *K*(*x*), one can pattern the sample into an array of Hall bar and fit the *K*-*x* relationship composed by the *K* data collected at different sites (*x*) using extraordinary Hall effect measurement^6^.

The variation of magnetic anisotropy in the track may lead to a space-coordinate dependent *φ*. However, the width of the domain wall is comparatively smaller than the length of the entire track, and the magnetic anisotropy can remain to be considered constant in the region of the domain wall. Therefore, *φ* is also approximately invariant in the range of the domain wall.

The Thiele equation for the domain wall motion is deduced by the Lagrangian approach. Let *l* be the Lagrangian density function and the expression can be written as follows^13^

3 $$l=E+({M}_{{\rm{S}}}/\gamma )\phi \dot{\theta }\,\sin \,\theta ,$$

where γ is the gyromagnetic ratio, $\dot{\theta }$ is the time derivative of *θ*, and *E* represents the total free energy density and can be written as

4 $$E={E}_{{\rm{e}}}+{E}_{{\rm{a}}}+{E}_{{\rm{d}}}+{E}_{{\rm{DM}}}.$$

*E*_e_, *E*_a_, *E*_d_, and *E*_DM_ are the exchange energy density, magnetic anisotropy energy density, demagnetization energy density, and free energy density from DMI, respectively. They are written as

5 $${E}_{{\rm{e}}}=A{\sum _{{\rm{i}}=x,y,z}|\nabla {m}_{{\rm{i}}}|}^{2},$$

where $\nabla$ is the Nabla symbol.

6 $${E}_{{\rm{a}}}=(K-\frac{1}{2}{\mu }_{0}{M}_{{\rm{S}}}^{2}){\sin }^{2}\theta ,$$

7 $${E}_{{\rm{d}}}=\frac{1}{2}{\mu }_{0}{N}_{{\rm{x}}}{M}_{{\rm{S}}}^{2}{\sin }^{2}\theta \,{\cos }^{2}\phi ,$$

8 $${E}_{{\rm{DM}}}=D[{m}_{{\rm{z}}}(\partial {m}_{{\rm{x}}}/\partial x)-{m}_{{\rm{x}}}(\partial {m}_{{\rm{z}}}/\partial z)],$$

and the demagnetization factor *N*_x_ is described by refs 9, 23

9 $${N}_{{\rm{x}}}={L}_{{\rm{z}}}\,\mathrm{ln}\,2/\pi {\rm{\Delta }},$$

where *L*_z_ is the thickness of Co layer.

For a non-conservative system, another dissipation density function *f*_d_ should be included to depict the dissipation^13^:

10 $${f}_{{\rm{d}}}=(\alpha {M}_{{\rm{S}}}/2{\rm{\gamma }}){[d\overrightarrow{m}/dt-({{\rm{\gamma }}}_{0}/\alpha ){H}_{{\rm{SO}}}(\overrightarrow{m}\times {\vec{e}}_{{\rm{y}}})]}^{2},$$

where *α* is the damping coefficient, and *H*_SO_ is the effective magnetic field due to SOT and is written as

11 $${H}_{{\rm{SO}}}={{\rm{\mu }}}_{{\rm{B}}}{\theta }_{{\rm{SH}}}J/{{\rm{\gamma }}}_{0}{\rm{e}}{M}_{{\rm{S}}}{L}_{{\rm{z}}},$$

where μ_B_, *θ*_SH_, *J*, and e are the Bohr magneton, spin Hall angle of the HM layer, current density, and charge of an electron, respectively. The parameter γ_0_ is related to γ by γ_0_ = μ_0_|γ|.

The Lagrangian (*L*) and Rayleigh dissipation function (*F*) were determined by integrating *l* and *f*_d_ with respect to the entire space region for the track. The Thiele equations are finally deduced using the Lagrange–Rayleigh equation:

12 $$\frac{\partial L}{\partial {q}_{i}}-\frac{d}{dt}(\frac{\partial L}{\partial {\dot{q}}_{i}})+\frac{\partial F}{\partial {\dot{q}}_{i}}=0,$$

where *q*_i_ represents the collective coordinate *q* or *φ* and *t* is time.

The domain wall motion in the track with magnetic anisotropy gradient is depicted by the following Thiele equations:

13 $$\begin{array}{rcl}(\alpha {M}_{{\rm{S}}}{I}_{{\rm{5}}}/\gamma )\dot{q}+({M}_{{\rm{S}}}{I}_{{\rm{8}}}/\gamma \sqrt{A})\dot{\phi } & = & -\,A(\partial {I}_{1}/\partial q)-a(\partial {I}_{2}/\partial q)\\  &  & -\,(b-\frac{1}{2}{\mu }_{0}{M}_{{\rm{S}}}^{2})(\partial {I}_{3}/\partial q)\\  &  & -\,D\,\cos \,\phi (\partial {I}_{4}/\partial q)\\  &  & -\,({\mu }_{0}{L}_{{\rm{z}}}{M}_{{\rm{S}}}^{2}\,{\cos }^{2}\,\phi \,\mathrm{ln}\,2/2\pi \sqrt{A})\\  &  & \times \,(\partial {I}_{8}/\partial q)\\  &  & -\,({M}_{{\rm{S}}}{\gamma }_{0}/\gamma ){H}_{{\rm{SO}}}{I}_{7}\,\cos \,\phi ,\end{array}$$

14 $$\begin{array}{rcl}(-{M}_{{\rm{S}}}{I}_{8}/\gamma \sqrt{A})\dot{q}+(\alpha {M}_{{\rm{S}}}{I}_{3}/\gamma )\dot{\phi } & = & D{I}_{4}\,\sin \,\phi +({\mu }_{0}{L}_{{\rm{z}}}{M}_{{\rm{S}}}^{2}(\mathrm{ln}\,2)\\  &  & \times \,\cos \,\phi \,\sin \,\phi /\pi \sqrt{A}){I}_{{\rm{8}}}\\  &  & +\,({M}_{{\rm{S}}}{\gamma }_{0}/\gamma ){H}_{{\rm{SO}}}{I}_{6}\,\sin \,\phi ,\end{array}$$

where *I*_1_–*I*_8_ are the integrals with the following formulas:

15 $$\begin{array}{rcl}{I}_{1} & = & {\int }_{-\frac{L{\rm{x}}}{{\rm{2}}}}^{\frac{L{\rm{x}}}{{\rm{2}}}}({\sin }^{2}\,\theta ){(\frac{\partial f}{\partial x})}^{2}\,{\rm{d}}x;{I}_{2}={\int }_{-\frac{L{\rm{x}}}{{\rm{2}}}}^{\frac{L{\rm{x}}}{{\rm{2}}}}x({\sin }^{2}\,\theta ){\rm{d}}x;{I}_{3}={\int }_{-\frac{L{\rm{x}}}{{\rm{2}}}}^{\frac{L{\rm{x}}}{{\rm{2}}}}{\sin }^{2}\,\theta \,{\rm{d}}x;\\ {I}_{4} & = & {\int }_{-\frac{L{\rm{x}}}{{\rm{2}}}}^{\frac{L{\rm{x}}}{{\rm{2}}}}(\frac{\partial f}{\partial x})\,\sin \,\theta \,{\rm{d}}x;{I}_{5}{\int }_{-\frac{L{\rm{x}}}{{\rm{2}}}}^{\frac{L{\rm{x}}}{{\rm{2}}}}({\sin }^{2}\,\theta ){(\frac{\partial f}{\partial q})}^{2}\,{\rm{d}}x;{I}_{6}={\int }_{-\frac{L{\rm{x}}}{{\rm{2}}}}^{\frac{L{\rm{x}}}{{\rm{2}}}}\sin \,\theta \,\cos \,\theta \,{\rm{d}}x;\\ {I}_{7} & = & {\int }_{-\frac{L{\rm{x}}}{{\rm{2}}}}^{\frac{L{\rm{x}}}{{\rm{2}}}}(\frac{\partial f}{\partial q})\,\sin \,\theta \,{\rm{d}}x;{I}_{8}={\int }_{-\frac{L{\rm{x}}}{{\rm{2}}}}^{\frac{L{\rm{x}}}{{\rm{2}}}}\sqrt{ax+b-\frac{1}{2}{\mu }_{0}{M}_{S}^{2}}({\sin }^{2}\,\theta )\,{\rm{d}}x.\end{array}$$

Here, *L*_x_ is the length of nanotrack, and

16 $$\begin{array}{rcl}f & = & (x-q)\sqrt{ax+b-\frac{1}{2}{\mu }_{0}{M}_{S}^{2}}/\sqrt{A};\\ \sin \,\theta  & = & \frac{2\,\exp \,(f)}{1+{[\exp (f)]}^{2}};\\ \cos \,\theta  & = & \frac{1-{[\exp (f)]}^{2}}{1+{[\exp (f)]}^{2}}.\end{array}$$

Equations (14) and (15) were solved using a 4^th^-order Runge–Kutta algorithm with a time step of 1 ps. The integrals *I*_1_–*I*_8_ were numerically evaluated using an adaptive Gauss–Kronrod quadrature. In the calculation, the nanotrack has the dimensions 1 μm (length) × 100 nm (width) × 0.6 nm (thickness). Typical magnetic parameters for PM Ta/CoFe bilayers were used^9^:*M*_S_, *b*, *A*, *D*, and *α* for CoFe are 7 × 10^5^ A/m, 4.8 × 10^5^ J/m^3^, 1 × 10^−11^ J/m, −0.05 mJ/m^2^, and 0.03, respectively. The spin-Hall angle (*θ*_SH_) for Ta is −0.11. The slopes for the anisotropy constant (*a*) are 0, 1 × 10^11^, 2 × 10^11^, and 3 × 10^11^ J/m^4^. A larger *a* cannot ensure effective perpendicular magnetic anisotropy throughout the entire track.

### Micro-magnetic simulation

The motion of the domain wall in the nanotrack with magnetic anisotropy gradient was also investigated by micro-magnetic simulation using the software “object-oriented micromagnetic framework” (OOMMF) with code including DMI^24^. The principle of the simulation is based on solving the Gilbert equation

17 $$\frac{\partial \overrightarrow{m}}{\partial t}=-\,{{\rm{\gamma }}}_{{\rm{0}}}\overrightarrow{m}\times {\overrightarrow{H}}_{{\rm{eff}}}+\alpha (\overrightarrow{m}\times \frac{\partial \overrightarrow{m}}{\partial t})+{{\rm{\gamma }}}_{{\rm{0}}}{H}_{{\rm{SO}}}(\overrightarrow{m}\times (\overrightarrow{\sigma }\times \overrightarrow{m})),$$

where ${\overrightarrow{H}}_{{\rm{eff}}}$ is the effective magnetic field derived from the free energy density:

18 $${\overrightarrow{H}}_{{\rm{eff}}}=-\,\frac{1}{{\mu }_{0}{M}_{{\rm{S}}}}(\frac{\delta E}{\delta \overrightarrow{m}}),$$

which includes the effective field from exchange, magnetic anisotropy, demagnetization, and DMI.

We consider a Ta/CoFe nanotrack whose shape and magnetic parameters are identical to that in numerical calculation. In current OOMMF software, it is not possible to generate magnetic anisotropy constants that change with *x* continuously. As an approximation to the case of *a* = 3 × 10^11^ J/m^4^ and *J* = ±5 × 10^11^ A/m^2^ in numerical calculation, we fabricated an anisotropy constant that is a piecewise constant function of *x*. This piecewise constant function of *K* can be expressed as follows,

19 $$K=\{\begin{array}{c}1\times {10}^{7}{\rm{J}}/{{\rm{m}}}^{3},(-500{\rm{n}}{\rm{m}},-450{\rm{n}}{\rm{m}})\\ {K}_{0},(-450{\rm{n}}{\rm{m}},(-450+{\rm{\Delta }}x){\rm{n}}{\rm{m}})\\ {K}_{0}+{\rm{\Delta }}K,((-450+{\rm{\Delta }}x){\rm{n}}{\rm{m}},(-450+2{\rm{\Delta }}x){\rm{n}}{\rm{m}})\\ {K}_{0}+2{\rm{\Delta }}K,((-450+2{\rm{\Delta }}x){\rm{n}}{\rm{m}},(-450+3{\rm{\Delta }}x){\rm{n}}{\rm{m}})\\ \cdots \cdots \cdots \cdots \\ {K}_{0}+N{\rm{\Delta }}K,((-450+N{\rm{\Delta }}x){\rm{n}}{\rm{m}},(-450+(N+1){\rm{\Delta }}x){\rm{n}}{\rm{m}})\\ 1\times {10}^{7}{\rm{J}}/{{\rm{m}}}^{3},(450{\rm{n}}{\rm{m}},500{\rm{n}}{\rm{m}}).\end{array}$$

The anisotropy constant close to the two ending points of the track (from −500 to −450 nm and from 450 to 500 nm) was set as large as 1 × 10^7^ J/m^3^ to pin boundary moments. The remaining nanotrack (*x* = −450 nm to *x* = 450 nm) was evenly divided into *N* + 1 steps with the size of Δ*x* for every step. The anisotropy constant varies from 3.45 × 10^5^ to 6.0 × 10^5^ J/m^3^ in accordance with an even step of Δ*K*. In our simulation, *N* includes 35, 17, and 9. Accordingly, Δ*x* includes 25 nm, 50 nm, and 100 nm, and Δ*K* includes 0.075 J/m^3^, 0.15 J/m^3^, and 0.3 J/m^3^, respectively.

## Results

**Figure 2:**
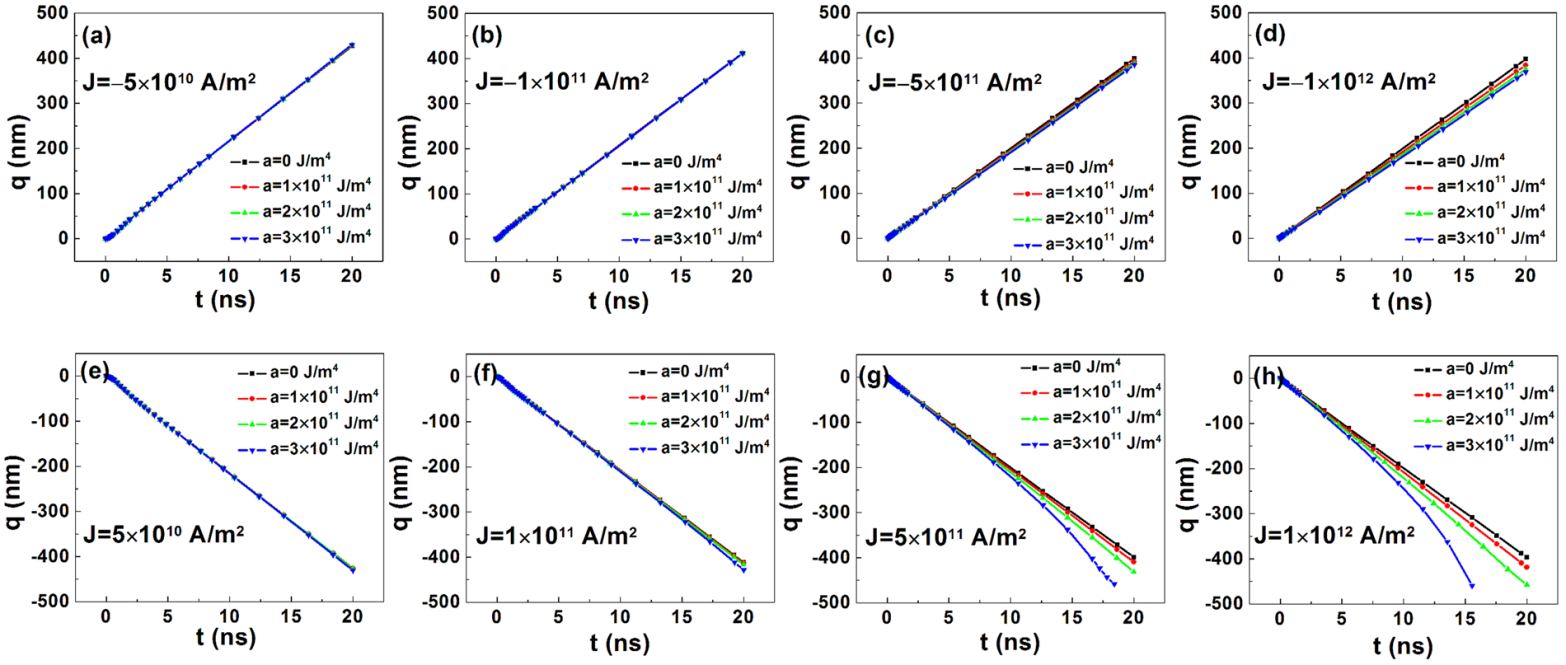
Change in time (*t*) of the central position of the domain wall (*q*) in the nanotracks with magnetic anisotropy gradient under current with different densities. The four figures in the first row show the *q*−*t* curves under negative current with the current density (*J*) of (**a**) −5 × 10^10^ A/m^2^, (**b**) −1 × 10^11^ A/m^2^, (**c**) −5 × 10^11^ A/m^2^, and (**d**) −1 × 10^12^ A/m^2^ respectively. The four figures in the second row show the *q*-*t* curves under positive current with the *J* of (**e**) 5 × 10^10^ A/m^2^, (**f**) 1 × 10^11^ A/m^2^, (**g**) 5 × 10^11^ A/m^2^, and (**h**) 1 × 10^12^ A/m^2^ respectively. The slopes for the anisotropy constant (*a*) are 0 (marked in black), 1 × 10^11^ (marked in red), 2 × 10^11^ (marked in green), and 3 × 10^11^ J/m^4^ (marked in blue).

Figure 2 shows the current-induced evolution of the central position of the domain wall in the nanotrack with magnetic anisotropy gradient. The negative (positive) current induces the motion of the domain wall towards the positive (negative) *x* direction, and this indicates that the domain wall moves along the direction of the moving election but against that of the current. This direction is consistent with that for negative *D* and negative *θ*_SH_^4, 9^.

When *a* = 0 J/m^4^, a linear *t*–*q* relationship is well satisfied except at the initial moving stage (*t* < 1 ns). It is also found that the *t*-*q* curves with negative *J* are symmetric to those with positive *J*. In other words, the domain wall moves towards opposite directions at the same speed with *J* of different signs. This result is consistent with the reported ones^3, 11, 13^ and is justified, since SOT which drives the domain wall to move is proportional to *J*, and it contributes to the motion of domain wall given that the gradient of magnetic anisotropy does not exist. As to the track with magnetic anisotropy gradient, we found that when *J* is as small as ±5 × 10^10^ A/m^2^, the gradient of the anisotropy constant has little effect on the motion of the domain wall. With the increase of *J*, it is clear that the gradient of the anisotropy constant affects the motion of the domain wall. Under negative *J*, the domain wall moves towards the direction of increasing anisotropy, and it moves slower with increasing *a*. In contrast, the domain wall moves faster towards the direction of decreasing anisotropy. In particular, unlike the uniform motion under negative *J*, the motion of the domain wall is accelerated under a strong positive *J* (1 × 10^12^ A/m^2^) when *a* is as large as 3 × 10^11^ J/m^4^.

**Figure 3:**
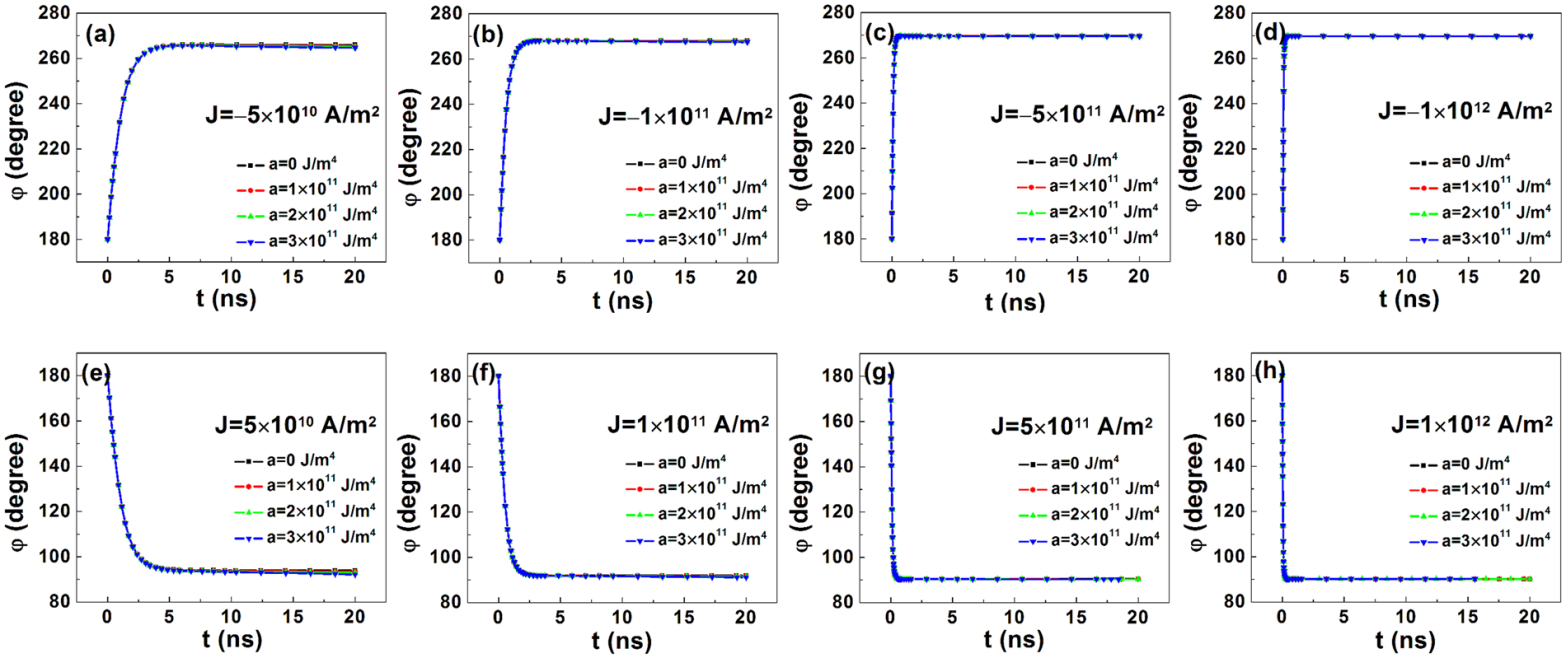
Time (*t*) dependence of the azimuthal angle (*φ*) of domain wall moments in nanotracks with magnetic anisotropy gradient under current with different densities. The four figures in the first row show that *φ*-*t* curves under negative current with respective current density (*J*) of (**a**) −5 × 10^10^ A/m^2^, (**b**) −1 × 10^11^ A/m^2^, (**c**) −5 × 10^11^ A/m^2^, and (**d**) −1 × 10^12^ A/m^2^. The four figures in the second row demonstrate that *φ*-*t* curves under positive current with respective *J* of (**e**) 5 × 10^10^ A/m^2^, (**f**) 1 × 10^11^ A/m^2^, (**g**) 5 × 10^11^ A/m^2^, and (**h**) 1 × 10^12^ A/m^2^. The slopes for the anisotropy constant (*a*) are 0 (marked in black), 1 × 10^11^ (marked in red), 2 × 10^11^ (marked in green), and 3 × 10^11^ J/m^4^ (marked in blue).

The gradual changes of the azimuthal angle *φ* under different *J* are depicted in Fig. 3. When *J* takes a negative value, the moments in domain wall rotate to approximately 270°. By contrast, the moments rotate to approximately 90° under positive *J*. These figures are consistent with the reported results^9^. When *J* is as small as ±5 × 10^10^ A/m^2^, the rotation is slower when compared with that under a stronger *J*, and *φ* is slightly smaller under a larger *a*. Under stronger current, the moments in the domain wall rotate very rapidly to their stable states, and the gradient of anisotropy has little effect on the final *φ*.

We have also investigated the *J*-dependent velocity for the current-induced motion of the domain wall, including the average velocity during the entire 15-ns moving process and the terminal velocity at the final moving stage.

**Figure 4:**
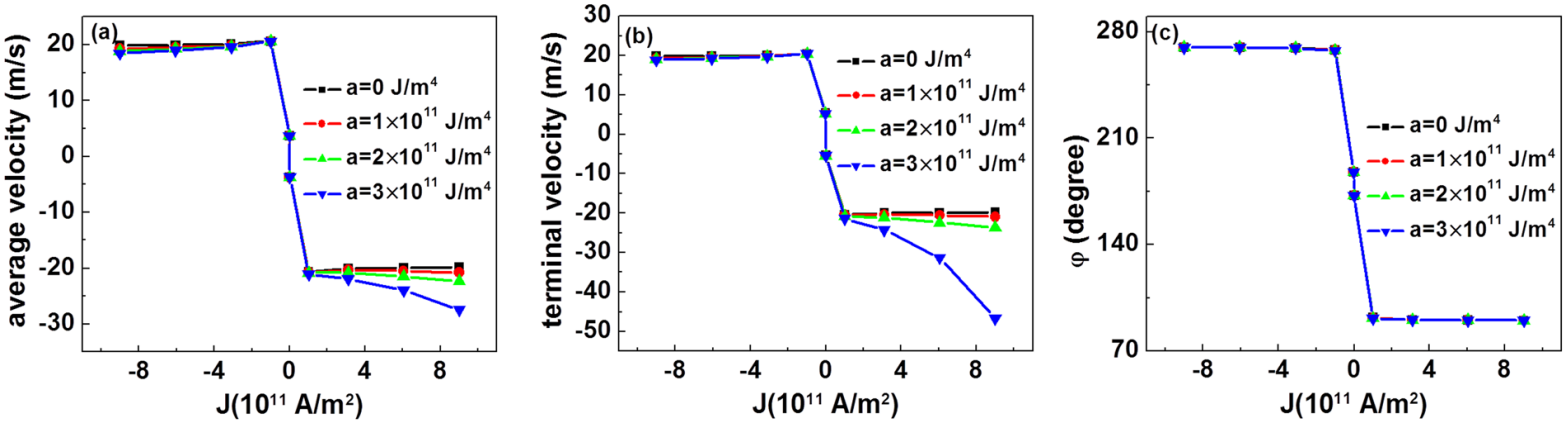
Current density dependence of the (**a**) average velocity, (**b**) terminal velocity, and (**c**) terminal azimuthal angle of domain wall moments in the nanotracks with magnetic anisotropy gradient.

As observed in Fig. 4(a) and (b), when *a* is 0 J/m^4^, both the average velocity and the terminal velocity increase very rapidly to around ±20 m/s when *J* increases to ±1 × 10^11^ A/m^2^. Such velocity is in accordance with the values reported for similar materials^9^. At higher current, however, both velocities exhibit a small decrease. A similar result of numerical calculation was reported^25, 26^. Under high current, the decrease of velocity is due to the transition of the domain-wall structure. At zero or very low current, the domain wall prefers a Néel-type structure due to the DMI, and the velocity of domain wall increases with the increase in *J*. At high current densities, to the contrary, under the action of very strong SOT, the domain-wall moments rotate towards the *y* axis, exhibiting a Bloch-type-like structure, as observed in Fig. 4(c). In this case, the velocity for the SOT-driven domain wall motion decreases^25, 26^. When *a* is non-zero, in the *J* range between 0 and ±1 × 10^11^ A/m^2^, the gradient of the anisotropy constant has little effect on either velocity. However, the gradient of anisotropy clearly affects the velocity of the domain wall for larger *J*. At negative current, the domain wall moves towards high anisotropy, and the velocity decreases with increasing *a*. Under positive current, the domain wall moves towards the end with low anisotropy. In this direction, the reduction of anisotropy greatly affects the velocity. Under a current of *J* = 1 × 10^12^ A/m^2^, when *a* increases from 0 to 3 × 10^11^ J/m^4^, the average velocity increases from approximately −20 m/s to approximately −30 m/s, and the terminal velocity is greatly enhanced from approximately −20 m/s to approximately −50 m/s. This increase in the terminal velocity is due to the non-linear *q*–*t* relationship shown in Fig. 2(h) under a large anisotropy slope. This result is important for applications because it indicates that the velocity of the domain wall can be more than doubled using anisotropy engineering, which is beneficial for enhancing the reading speed of domain-wall-type magnetic information storage media.

**Figure 5:**
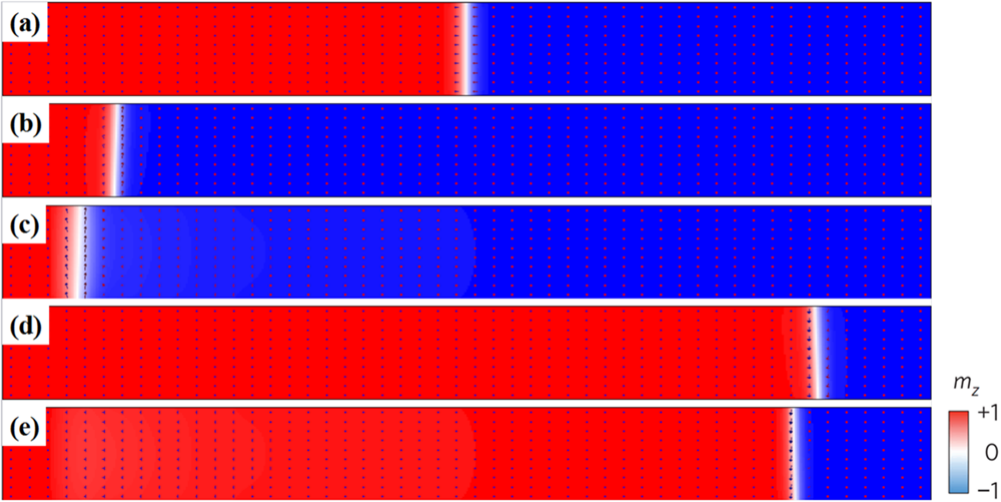
Micromagnetic configuration of SOT-induced domain wall motion in the 1000-nm-length track with piecewise anisotropy constant. (**a**) Snapshot of initial structure of a left-handed (*D* < 0) up-down domain wall. Snapshot of the domain wall moving after 20 ns under a current of *J = *5 × 10^11^ A/m^2^ in a track with (**b**) constant anisotropy (*a* = 0 J/m^4^) and (**c**) sloped anisotropy (*a* = 3 J/m^4^). Snapshot of the domain wall moving after 20 ns under a current of *J* = −5 × 10^11^ A/m^2^ in a track with (**d**) constant anisotropy (*a* = 0 J/m^4^) and (**e**) sloped anisotropy (*a* = 3 J/m^4^).

To justify the numerical calculation on the SOT-driven motion of the domain wall in a track with gradient of magnetic anisotropy, we have run related micro-magnetic simulation as reference. The results of micro-magnetic simulation on the piecewise constant function of *K* with *N* = 17, Δ*x* = 50 nm, and Δ*K* = 0.15 J/m^3^ in Eq. (19) are shown in Fig. 5. We selected an up-down domain wall structure as an example. In the initial state, the domain wall has a typical Néel-type structure with its moments pointing to the up side (+*z*) because of the negative *D* (Fig. 5(a)). Under positive current, the domain wall moves to the left side, and the *φ* of the moments in the domain wall is close to 90° (Fig. 5(b) and (c)). The reduction of the anisotropy constant clearly enhances the moving velocity and the width of the domain wall. In contrast, applying a positive current leads to the motion of domain wall towards the right side, where the higher anisotropy reduces the moving velocity of the domain wall, and the arrows in Fig. 5(d) and (e) indicate that *φ* is close to 270°. These results are consistent with those of the numerical calculations.

**Figure 6:**
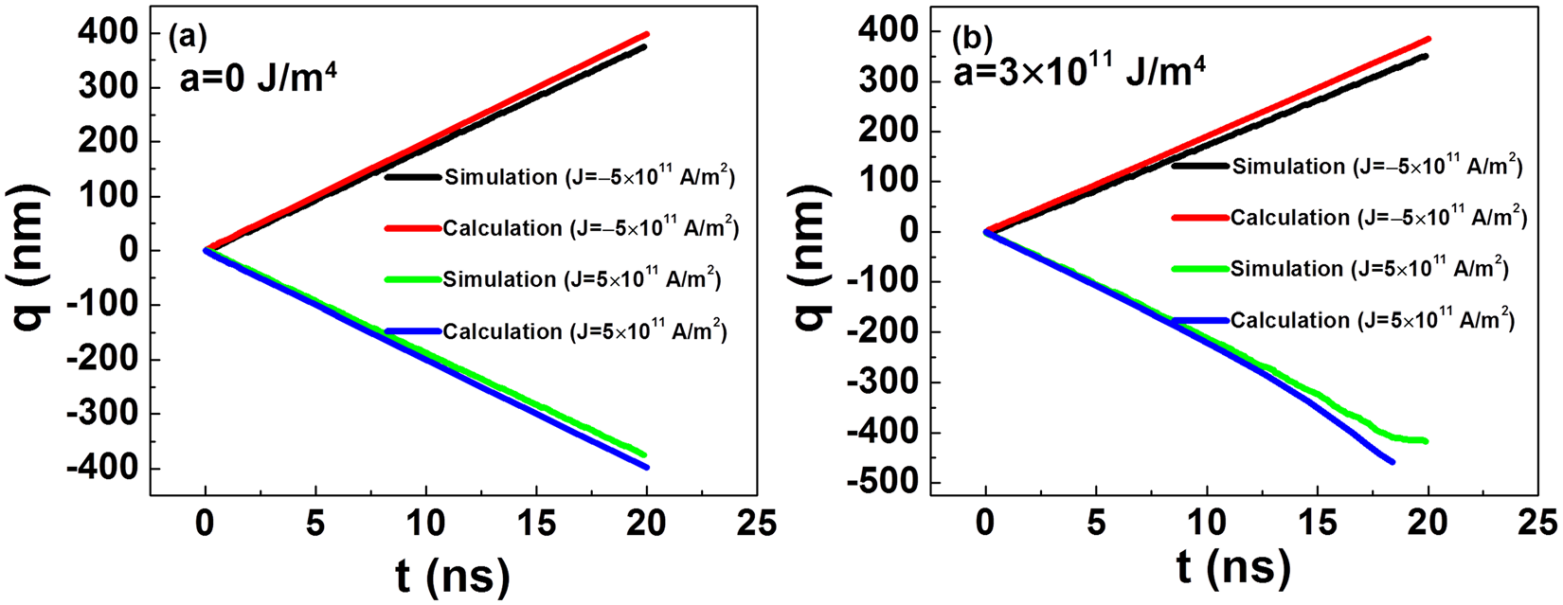
Comparison of numerical calculation and micro-magnetic simulation results for (**a**) *a* = 0 J/m^4^ and (**b**) *a* = 3 J/m^4^.

In Fig. 6, the simulation results are quantitatively compared with those of the numerical calculation. The micro-magnetic simulation and numerical calculation yield similar results. The difference between them is small and reasonable. This difference can be attributed to the following reasons: (1). Unlike the numerical calculation, in the simulation the changes of the anisotropy constant against *x* satisfies a piecewise constant function instead of a linear function. Therefore, the result approximation between the numerical calculation and the simulation for a small step size is well grounded. In addition to the step size of 50 nm shown in Figs 5 and 6, we also ran the simulation for the step sizes of 25 nm and 100 nm. The result (not shown) indicates that the difference of simulation results among different step sizes becomes negligible if the step size is 50 nm or smaller. Therefore, the step size of 50 nm is small enough for the simulation. Nevertheless, difference between the calculation and the simulation is still evident as shown in Fig. 6, and this difference is caused by the following factors. (2). In the numerical calculation, we do not consider the small tilting of the entire domain wall because of the weak DMI^9^. However, this small tilting can be observed in the simulation results (Fig. 5(b–e)). This tilting of domain wall may also reduce the velocity of domain wall motion as compared with that without the tilting, and this is consistent with the previous results^9, 13^. For a sample with higher DMI, such as Pt/CoFe, the tilting of the domain wall cannot be neglected because of the competition between the DMI and demagnetization energy of the domain wall^9, 13^. In this case, the tilting angle of the domain wall must be contemplated in numerical calculations to avoid errors. (3). In the simulation, the moments which are close to the boundaries are pinned by strong anisotropy, and this may act as a barrier to repel the domain wall when it is close to the end, resulting in the decrease of velocity.

## Discussion

The current-driven motion of DW is dominated by the conjunctive action of several factors. For example, Li *et al*. discovered a screw-pitch effect (the combined motion of precession of DW moments and moving of DW center) as a result of the conjunct action of Gilbert damping and spin transfer torque (STT)^27^. In the SOT-induced DW motion, the DW is driven to move under the combined action of torque-like SOT (Equation 17), the gradient of anisotropy, and other contributions of free energy. To clarify the mechanism, we focus on the SOT and the gradient of magnetic anisotropy constants.

**Figure 7:**
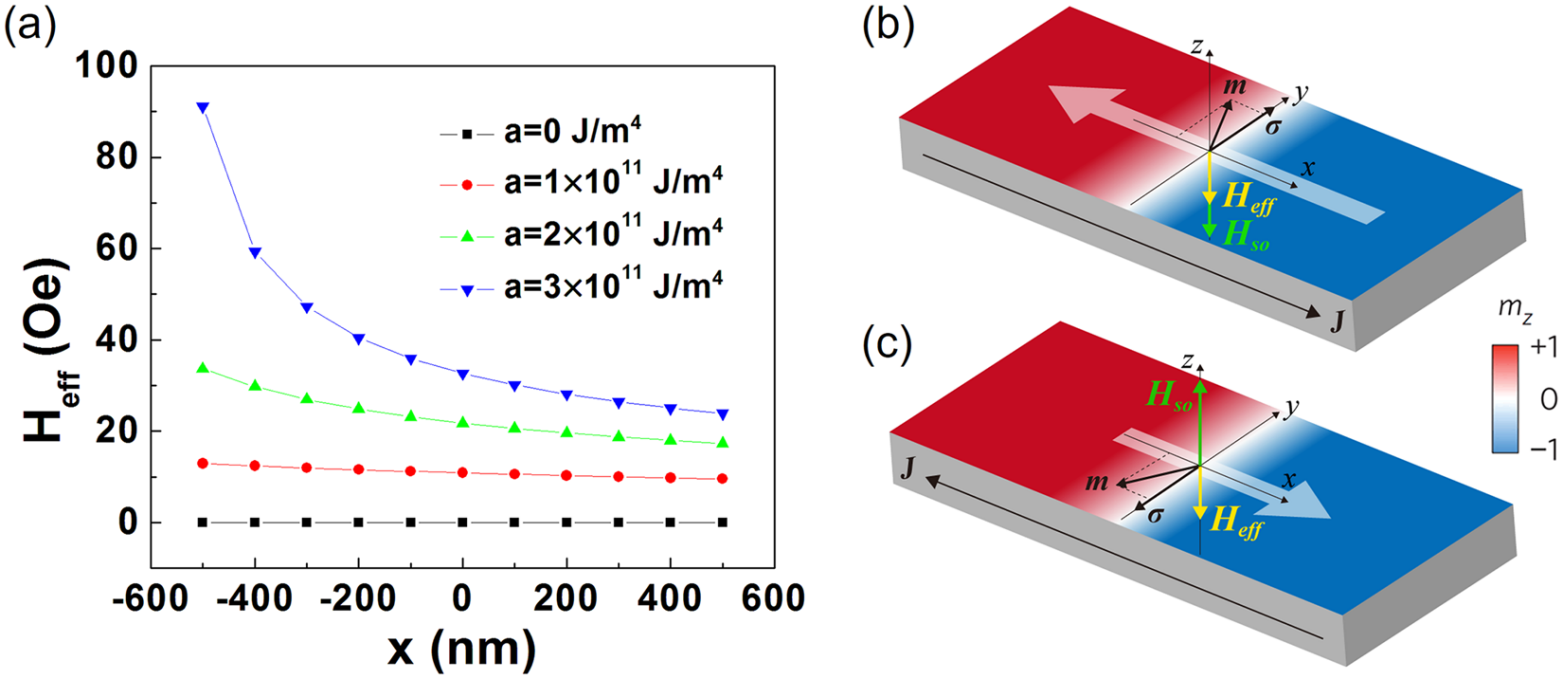
(**a**) The effective field due to the magnetic anisotropy gradient and its variation with *x*. The slopes for *K*(*x*) function of (*a*) are 0 (marked in black), 1 × 10^11^ (marked in red), 2 × 10^11^ (marked in green), and 3 × 10^11^ J/m^4^ (marked in blue). (**b**) and (**c**). Schematic of DW motion driven by both effective fields contributed from SOT (*H*_SO_) and the gradient of magnetic anisotropy (*H*_eff_). When current is along (**b**) + x direction, both *H*_SO_ and *H*_eff_ are in the −*z* direction, while when current is along (**c**) −x direction, *H*_SO_ and *H*_eff_ have opposite directions.

According to the Gilbert equation (Eq. 17), the effective field for SOT (***H***_SO_) is written as:

20 $${\overrightarrow{H}}_{{\rm{SO}}}=\frac{{{\rm{\mu }}}_{{\rm{B}}}{\theta }_{{\rm{SH}}}J}{{{\rm{\gamma }}}_{0}{\rm{e}}{M}_{{\rm{S}}}{L}_{{\rm{z}}}}(\overrightarrow{m}\times \overrightarrow{\sigma }),$$

where μ_B_, γ_0_, *M*_S_, *L*_z_, and *J* are all positive (The variation of direction of current density changes the direction of ***σ***); *θ*_SH_ and e are both negative. As to a negative *θ*_SH_, when current is along +*x* (−*x*) direction, ***σ*** is along +*y* (−*y*) direction, and the angle (*φ*) of moment of DW is between 90° and 180° (180° and 270°) (Fig. 7(b) and (c) and Fig. 3). Therefore, when electrons move towards −*x* (+*x*) direction, the ***H***_SO_ is along −*z* (+*z*) direction, pushing the DW to move in the −*x* (+*x*) direction. In the track with constant magnetic anisotropy, the currents with the same strength and opposite signs offer the effective fields with identical strength and opposite directions, driving the domain wall to move in opposite directions at the same speed^3, 11, 23, 25, 28, 29^.

When the magnetic anisotropy constant varies linearly with *x*, the change of domain wall energy against *x* can be phenomenologically converted into effective field (*H*_eff_) expressed as^21^:

21 $${H}_{{\rm{eff}}}=\frac{1}{2{M}_{{\rm{S}}}}(\frac{\partial \sigma }{\partial x}),$$

where *σ* means the domain wall energy density, and its *x*-dependent term is ${\rm{4}}\sqrt{A{K}_{{\rm{eff}}}}$ $({K}_{{\rm{eff}}}=K-\frac{1}{2}{\mu }_{0}{M}_{{\rm{S}}}^{2}=ax+b-\frac{1}{2}{\mu }_{0}{M}_{{\rm{S}}}^{2})$ ^21^. Therefore,

22 $${H}_{{\rm{eff}}}=\frac{a\sqrt{A}}{{M}_{{\rm{S}}}\sqrt{ax+b-\frac{1}{2}{\mu }_{0}{M}_{{\rm{S}}}^{2}}}.$$

The variation of *H*_eff_ with *x* for different *a* is shown in Fig. 7(a). First of all, it can be observed that the constant magnetic anisotropy (*a* = 0 J/m^4^) means zero *H*_eff_. Moreover, *H*_eff_ goes up monotonously with the increase of *a* and the decrease of *x*. In particular, *H*_eff_ increases significantly with the decrease of *x* when the value of *a* reaches as large as 3 × 10^11^ J/m^4^. This ***H***_eff_ is along −*z* direction so that the domain can move towards the track end with lower anisotropy (−*x* direction)^21, 30^.

At positive current, the domain wall moves towards negative *x* direction with weak magnetic anisotropy. In this case, both ***H***_eff_ and ***H***_SO_ share the same direction (Fig. 7(b)). As a result, the velocity of domain wall is larger than that for *a* = 0 J/m^4^. As to a large gradient of magnetic anisotropy constant (*a* = 3 J/m^4^), the *H*_eff_ is significantly enhanced with a decreasing *x*. Therefore, the domain wall moves at an ever increased rate. At negative current, the domain wall moves towards the positive *x* direction with stronger magnetic anisotropy energy under both ***H***_eff_ and ***H***_SO_. The direction of ***H***_eff_ is opposite to that of ***H***_SO_ (Fig. 7(c)). Therefore, the velocity is comparatively reduced when compared to that for *a* = 0 J/m^4^. Additionally, it is also observed that in regions with large positive *x*, *H*_eff_ can be approximately seen as a constant field, which ensures the uniform motion of the domain wall.

In a word, the symmetry of DW motion driven by ***H***_SO_ is broken by the additional ***H***_eff_ due to the gradient of magnetic anisotropy. It is interesting to see that similar magnetic field-induced symmetry breaking exists widely in nature, such as the magnetic field-induced asymmetric Josephson energy for a Josephson ratchet composed by a *φ* Josephson junction and a ferromagnetic barrier^31^. Additionally, besides the Thiele equations used in this paper, the current-driven motion of domain wall can also be investigated by some other methods. For example, Li *et al*. derived an effective Newton’s equation for depicting the motion of a rigid-body DW driven by STT in a ferromagnetic nanowire with DMI^32^. The SOT-driven motion of DW in a track with the gradient of magnetic anisotropy using this method deserves further investigated.

## Summary

In summary, using numerical calculations based on the collective coordinate model and micro-magnetic simulation, we investigated the SOT-induced motion of a domain wall in a nanotrack with weak DMI and magnetic anisotropy gradient. The domain wall exhibits a Néel-type structure due to the DMI, and the structure transforms into a Bloch-type-like one under SOT. The velocity of the domain wall motion is manipulated by the variation of the magnetic anisotropy constant along the track. The enhanced magnetic anisotropy constant acts as a barrier to hinder domain wall motion; however, reducing the magnetic anisotropy constants pushes the domain wall to move at a substantially higher speed. When the current density is 1 × 10^12^ A/m^2^ and the slope for the space-variation of the anisotropy constant reaches 3 × 10^11^  J/m^4^, the velocity can be more than doubled compared with that in the track with uniform anisotropy constant. The divergence between positive and negative *J* on the domain wall motion is attributed to the combined action of SOT and the *x-*dependent effective magnetic field which derives from the magnetic anisotropy gradient.
