# Author Correction: Spin-orbit-torque-induced magnetic domain wall motion in Ta/CoFe nanowires with sloped perpendicular magnetic anisotropy

**DOI:** 10.1038/s41598-018-25613-3

**Published:** 2018-05-03

**Authors:** Yue Zhang, Shijiang Luo, Xiaofei Yang, Chang Yang

**Affiliations:** 1 School of Optical and Electronic Information, Huazhong University of Science and Technology, Wuhan, 430074 PR China; 2 Department of Physics and Astronomy, Johns Hopkins University, Baltimore, MD 21218 USA; 3 Department of Mathematics, Harbin Institute of Technology, Harbin, 150001 PR China

Correction to: *Scientific Reports* 10.1038/s41598-017-02208-y, published online 17 May 2017

This Article contains typographical errors in Equation (8), where

$${E}_{{\rm{DM}}}=D[{m}_{{\rm{z}}}(\partial {m}_{{\rm{x}}}/\partial x)-(\partial {m}_{{\rm{z}}}/\partial z)]$$

should read:

$${E}_{{\rm{DM}}}=D[{m}_{{\rm{z}}}(\partial {m}_{{\rm{x}}}/\partial x)-(\partial {m}_{{\rm{z}}}/\partial x)]$$

In addition, equation (15)

$$\begin{array}{ll}{I}_{1}={\int }_{-\frac{L{\rm{x}}}{2}}^{\frac{L{\rm{x}}}{2}}({\sin }^{2}\,\theta ){(\frac{\partial f}{\partial x})}^{2}{\rm{d}}x; & {I}_{2}={\int }_{-\frac{L{\rm{x}}}{2}}^{\frac{L{\rm{x}}}{2}}x({\sin }^{2}\,\theta ){\rm{d}}x;\\ {I}_{3}={\int }_{-\frac{L{\rm{x}}}{2}}^{\frac{L{\rm{x}}}{2}}{\sin }^{2}\,\theta \,{\rm{d}}x; & {I}_{4}={\int }_{-\frac{L{\rm{x}}}{2}}^{\frac{L{\rm{x}}}{2}}(\frac{\partial f}{\partial x})\,\sin \,\theta \,{\rm{d}}x;\\ {I}_{5}{\int }_{-\frac{L{\rm{x}}}{2}}^{\frac{L{\rm{x}}}{2}}({\sin }^{2}\,\theta ){(\frac{\partial f}{\partial q})}^{2}{\rm{d}}x; & {I}_{6}={\int }_{-\frac{L{\rm{x}}}{2}}^{\frac{L{\rm{x}}}{2}}\sin \,\theta \,\cos \,\theta \,{\rm{d}}x;\\ {I}_{7}={\int }_{-\frac{L{\rm{x}}}{2}}^{\frac{L{\rm{x}}}{2}}(\frac{\partial f}{\partial q})\,\sin \,\theta \,{\rm{d}}x; & {I}_{8}={\int }_{-\frac{L{\rm{x}}}{2}}^{\frac{L{\rm{x}}}{2}}\sqrt{ax+b-\frac{1}{2}{\mu }_{0}{M}_{S}^{2}}({\sin }^{2}\,\theta )\,{\rm{d}}x\end{array}$$

should read:

$$\begin{array}{ll}{I}_{1}={\int }_{-\frac{L{\rm{x}}}{2}}^{\frac{L{\rm{x}}}{2}}({\sin }^{2}\,\theta ){(\frac{\partial f}{\partial x})}^{2}{\rm{d}}x; & {I}_{2}={\int }_{-\frac{L{\rm{x}}}{2}}^{\frac{L{\rm{x}}}{2}}x({\sin }^{2}\,\theta )\,{\rm{d}}x;\\ {I}_{3}={\int }_{-\frac{L{\rm{x}}}{2}}^{\frac{L{\rm{x}}}{2}}{\sin }^{2}\,\theta \,{\rm{d}}x; & {I}_{4}={\int }_{-\frac{L{\rm{x}}}{2}}^{\frac{L{\rm{x}}}{2}}(\frac{\partial f}{\partial x})\,\sin \,\theta \,{\rm{d}}x;\end{array}$$

$$\begin{array}{ll}{I}_{5}={\int }_{-\frac{L{\rm{x}}}{2}}^{\frac{L{\rm{x}}}{2}}({\sin }^{2}\,\theta ){(\frac{\partial f}{\partial q})}^{2}{\rm{d}}x; & {I}_{6}={\int }_{-\frac{L{\rm{x}}}{2}}^{\frac{L{\rm{x}}}{2}}\sin \,\theta \,\cos \,\theta \,{\rm{d}}x;\\ {I}_{7}={\int }_{-\frac{L{\rm{x}}}{2}}^{\frac{L{\rm{x}}}{2}}(\frac{\partial f}{\partial q})\,\sin \,\theta \,{\rm{d}}x; & {I}_{8}={\int }_{-\frac{L{\rm{x}}}{2}}^{\frac{L{\rm{x}}}{2}}\sqrt{ax+b-\frac{1}{2}{\mu }_{0}{M}_{S}^{2}}({\sin }^{2}\,\theta )\,{\rm{d}}x\end{array}$$

